# An Update on Temporal Bone Paragangliomas

**DOI:** 10.1007/s11864-023-01127-7

**Published:** 2023-08-09

**Authors:** Shixun Zhong, Wenqi Zuo

**Affiliations:** Department of Otolaryngology, The First Affiliated Hospital of Chongqing Medical University, 1 Youyi Road, Yuzhong District, Chongqing, 400016 China

**Keywords:** Temporal bone, Paraganglioma, Surgical treatment, Radiotherapy

## Abstract

Temporal bone paragangliomas (TBPs) are indolent, classically benign and highly vascular neoplasms of the temporal bone. There are two types of TBPs, tympanomastoid paragangliomas (TMPs) and tympanojugular paragangliomas (TJPs). The most common symptoms are hearing loss and pulsatile tinnitus. Diagnostic workup, besides conventional physical and laboratory examinations, includes biochemical testing of catecholamine and genetic testing of SDHx gene mutations as well as radiological examination. Although surgery is traditionally the mainstay of treatment, it is challenging due to the close proximity of tumor to critical neurovascular structures and thus the high risk of complications, especially in patients with advanced lesions. Radiotherapy and active surveillance have been increasingly recommended for selected patients. Decision on treatment should be made comprehensively. Curative effect depends on various factors. Long-term follow-up with clinical, laboratory, and radiological examinations is essential for all patients.

## Introduction

Temporal bone paragangliomas (TBPs) are highly vascular and usually benign neoplasms of the temporal bone. They are the second most common paragangliomas of the head and neck, next to carotid body tumor, accounting for 20–30% of head and neck paragangliomas (HNPGLs) [1, 2]. A female predominance has been reported [3, 4]. The average age at presentation is the fifth decade of life [5, 6]. The annual incidence of glomus jugular tumors has been reported to be about 0.07 per 100,000 per year or 1 case per 1.3 million people [7].

TBPs are either sporadic or familial tumors. Approximately 40% of HNPGLs are caused by germline mutations, most commonly mutations in the succinate dehydrogenase gene SDHC, SDHD, SDHB, and SDHAF2 [8–10]. The patients with a positive family history, preceding pheochromocytoma, multifocal paragangliomas, malignant paragangliomas, and early presenting age (<50 years of age) may have higher incidence of germline mutations [8, 11]. Nevertheless, recent study showing tympanojugular paragangliomas (TJPs) in females are less associated with germline SDHx mutations suggests a distinct mechanism of tumorigenesis other than SDHx mutations [3].

## Classification

The TBPs originating from the tympanic branch of glossopharyngeal nerve (Jacobson’s nerve) and the auricular branch of vague nerve (Arnold’s nerve) are tympanomastoid paragangliomas (TMPs), and those originating from the paraganglia at the adventitia of the dome of the jugular bulb are tympanojugular paragangliomas (TJPs). A few patients present with multicentric tumors including carotid body tumors, vagal paragangliomas, or PGLs at other sites, accounting for 10–20% of all HNPGLs [12, 13].

To date, the most commonly used classification for TBPs is the Fisch classification which classifies TBPs into classes A, B, C, and D on the basis of location and extension of tumors [14] (Table 1). Prasad et al. and Shin et al. further subclassify classes A and B into A1 and A2 and B1, B2, and B3, respectively, and adds class V to include tumor involving vertebral artery [12, 15]. Another frequently used grading system is the Glasscock–Jackson classification [16], which does not differentiate a TJP from a TMP (Table 2).

**Table 1:** Fisch classification of temporal bone paragangliomas [14]

Class A (glomus tympanicum)	Limited to mesotympanum
Class B (glomus hypotympanicum)	Limited to hypotympanum, mesotympanum, and mastoid without erosion of jugular bulb
Class C	Involvement and destruction of infralabyrinthine and apical compartments Subclassification by degree of carotid canal erosion
C1	No invasion of carotid; destruction of jugular bulb/foramen
C2	Invasion of vertical carotid canal between foramen and bend
C3	Invasion along horizontal carotid canal
C4	Invasion of foramen lacerum and along carotid into cavernous sinus
Class D	Intracranial extension (De, extradural; Di, intradural)
De1	Up to 2-cm dural displacement
De2	More than 2-cm dural displacement
Di1	Up to 2-cm intradural extension
Di2	More than 2-cm intradural extension

**Table 2:** Glasscock-Jackson classification of glomus tumors [16]

Glomus tympanicum	
I	Small mass limited to promontory
II	Tumor completely filling middle ear space
III	Tumor filling middle ear and extending into the mastoid
IV	Tumor filling middle ear, extending into the mastoid or through tympanic membrane to fill the external auditory canal; may extend anterior to carotid
Glomus jugulare	
I	Small tumor involving jugular bulb, middle ear, and mastoid
II	Tumor extending under internal auditory canal; may have intracranial canal extension (ICE)
III	Tumor extending into petrous apex; may have ICE
IV	Tumor extending beyond petrous apex into clivus or infratemporal fossa; may have ICE

## Clinical features

**Fig. 1:**
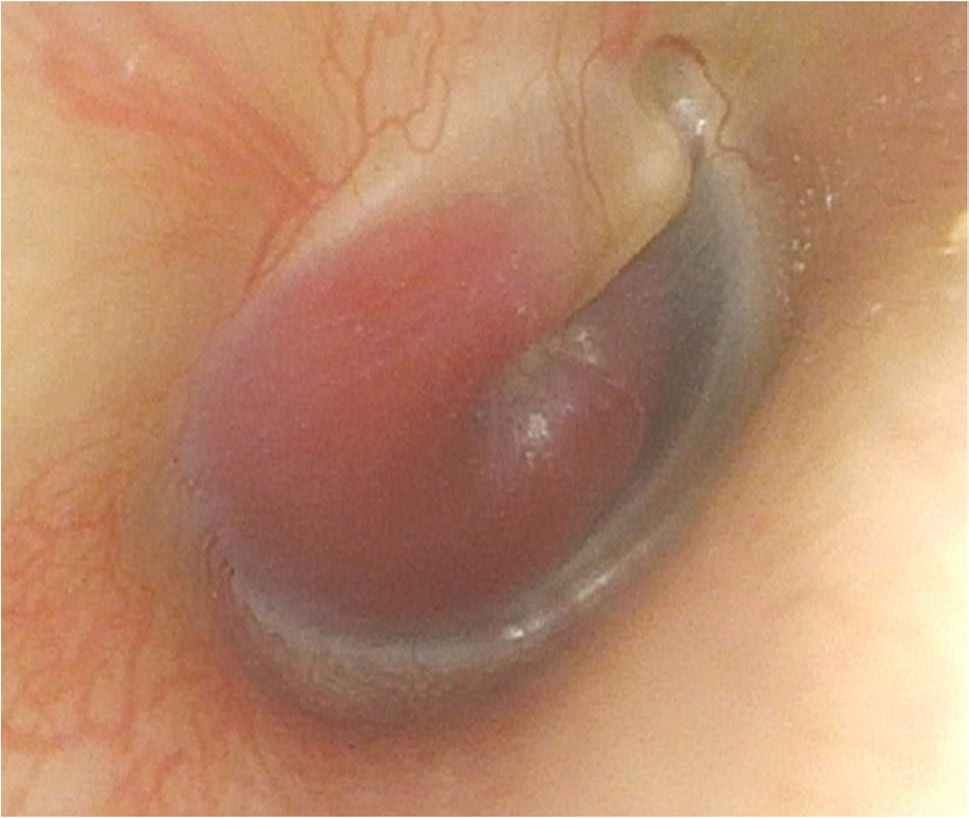
Otoscopic examination showing a tympanomastoid paraganglioma behind an intact tympanic membrane.

The most common symptoms of TBPs are hearing loss and pulsatile tinnitus. Hearing loss can be either conductive, sensorineural, or mixed, of which conductive hearing loss is more common. Some patients may present with chronic bloody otorrhea, vertigo, or facial paralysis. Large tumors may cause dysfunction of cranial nerves(CN) IX–XII, of which CN IX and X are most commonly affected, and thus may present with hoarseness, dysarthria, dysphagia, shoulder weakness, and so on [17–19]. Few patients have even CN IV–VI deficits. Prasad et al. [12] reported that almost half patients with TJPs (84/184) suffered from at least one CN deficit, and 56.2% tumors had intracranial extension. In patients with late lesions, increased intracranial pressure caused by brainstem compression and fourth ventricle effacement may result in headache and vomiting. Although most of the TBPs are nonsecretory, a few tumors do secrete catecholamines (1~8%) which may cause symptoms of sympathetic overactivity such as tachycardia, hypertension, flushing, and perspiration [20, 21]. The typical sign of TBPs under otoscope is a purple, pulsating mass behind the eardrum which usually blanches on pneumatic otoscopy (Brown’s sign) (Fig. 1). Once the tumor erodes the tympanic membrane, a pulsating mass can be found in the ear canal and middle ear.

Malignancy has been shown in a small portion of HNPGLs [22, 23]. Unlike for most tumors, however, there are no molecular, cellular, or histopathologic diagnostic criteria to date to accurately define malignant paragangliomas. Malignancy can only be diagnosed once metastasis to nonneuroendocrine tissue is identified [24, 25]. Metastasis is defined as PGLs in areas where normally paraganglia does not occur, in other words, as the presence of chromaffin in non-chromaffin tissue. The most common locations of metastasis are cervical lymph nodes and distant organs such as the lung, bone, and liver. Metastatic disease may develop years after the initial presentation of HNPGLs. SDHB mutated tumors have the highest rate of metastasis(30 to 50%) with poor overall survival [26]. Other factors associated with high risk of metastasis include younger age, secretory tumors, and rapidly increasing size [27, 28].

Nevertheless, since the 4th edition of the WHO classification of endocrine and neuroendocrine tumors, paragangliomas have no longer been classified as benign and malignant because any lesion can have metastatic potential and there are no clear-cut features that can predict metastatic behavior [29]. In addition, it must be kept in mind that multiple lesions do not always represent metastasis as they may be multifocal primary tumors too.

## Diagnostic workup

In addition to conventional laboratory tests and imaging examinations, the following special procedures must be taken into account before any treatment decision is made for the TBPs.

### Biochemical testing

The catecholamine levels should be examined for patients with such symptoms as tachycardia, hypertension, flushing, and perspiration to clarify whether the TBPs are secretory tumors or not. Catecholamines and their metabolites can be measured in both plasma and 24-h urinary collection. TBPs do not secrete epinephrine because the phenylethanolamine N-methyltransferase converting norepinephrine to epinephrine is confined to the adrenal medulla [30]. For secretory tumors with norepinephrine excess, preoperative management by endocrinologist is necessary because alpha adrenergic blockade is generally required to minimize perioperative complications. Nonselective α-blockers (e.g., phenoxybenzamine) or selective α-blockers (e.g., doxazocin) should be prescribed at least 7–14 days preoperatively so as to allow adequate time to normalize blood pressure and heart rate.

### Genetic testing

Studies have demonstrated that genetic testing is an effective tool for earlier detection of tumors as well as predicting the incidence of metastasis [31]. Early intervention can then be adopted to obtain a better outcome. Genetic counseling of probands and their families is an essential part of the management of paragangliomas. The Endocrine Society and European Society of Endocrinology recommend that all patients with HNPGLs should be screened for SDHx mutations because 40% of patients with PPGLs have germline mutations and about 25% of patients with SDHB mutations have metastasis [32, 33]. Due to the high sensitivity and specificity of SDHB immuno-histochemical evaluation, the new WHO classification recommends immuno-histochemical testing of SDHB in all HNPGLs [34].

### Radiology

**Fig. 2:**
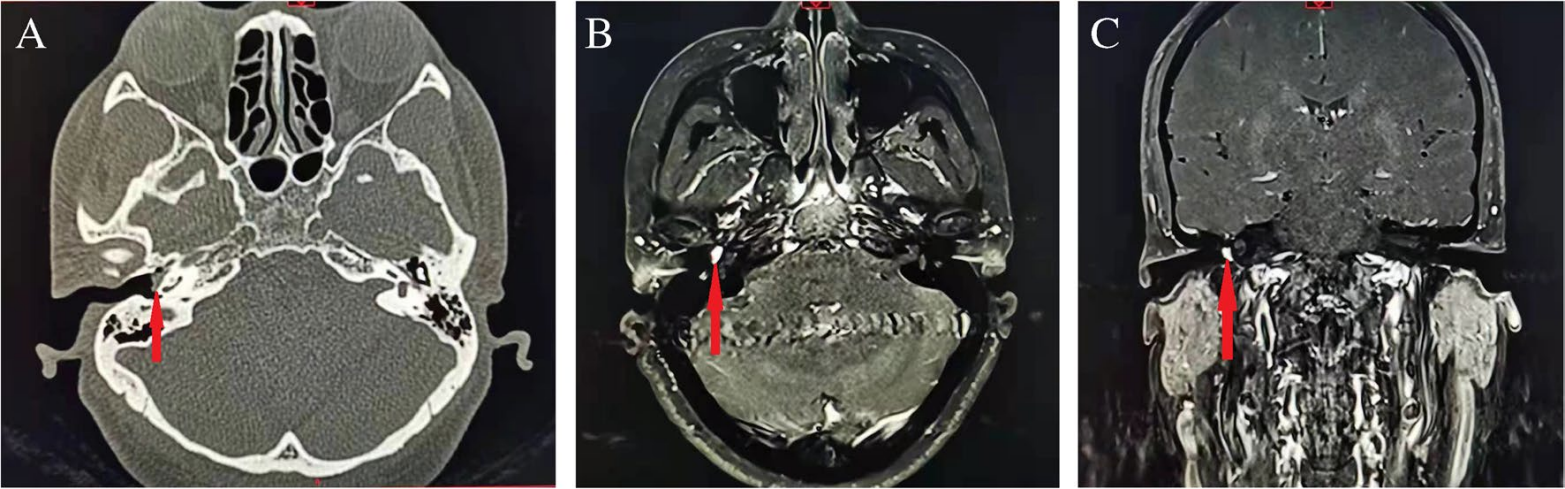
A tympanomastoid paraganglioma in the right tympanic cavity. **A** Axial CT scan. **B**, **C** Axial (**B**) and coronal (**C**) T1-weighted contrast-enhanced MRI. Arrows indicate a mass in the right middle ear lateral to the cochlear promontory.

**Fig. 3:**
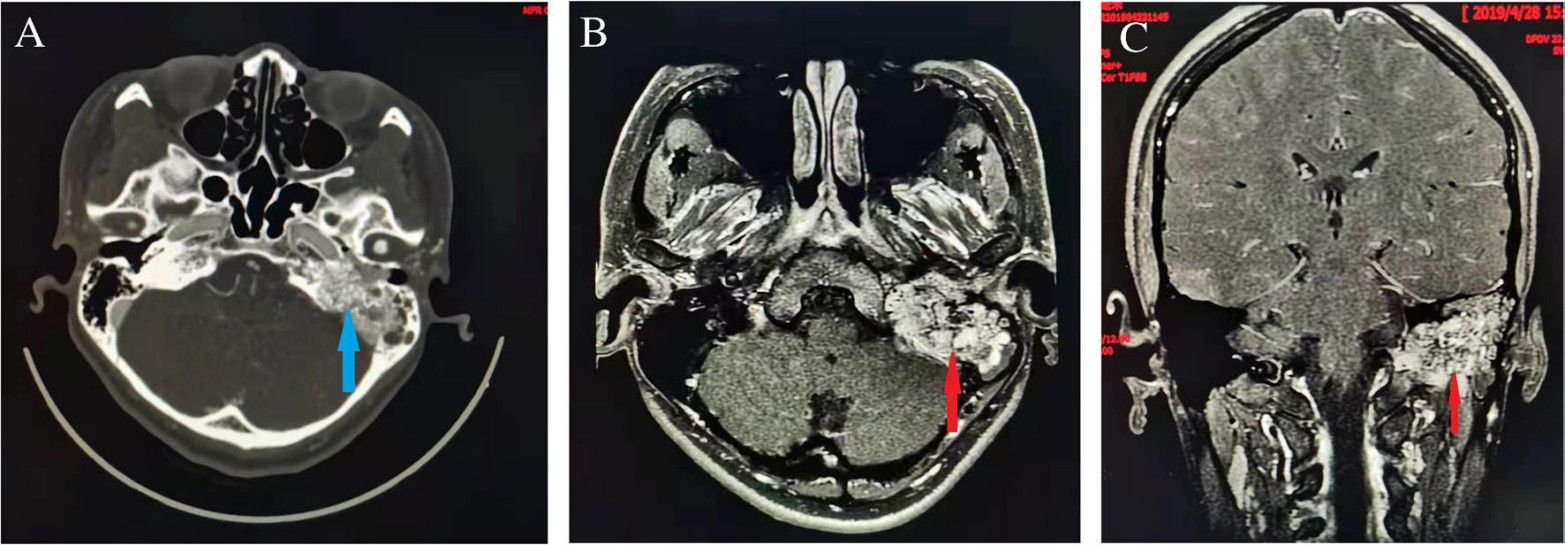
A tympanojugular paraganglioma at the left jugular foramen. **A** Axial CT scan. **B**, **C** Axial (**B**) and coronal (**C**) T1-weighted contrast-enhanced MRI. Blue arrow indicates the bony destruction with a moth-eaten appearance. Red arrows indicate the tumor with a salt-and-pepper appearance.

Radiological examination should be performed for all patients with suspected lesions. CT and MRI with or without enhancement are essential for diagnosis with similar sensitivity (80–90%) and specificity (90%)^1^. CT is useful to show bony erosion of temporal bone and skull base, with a typical “moth-eaten” pattern in case of TJP (Figs. 2 and 3). Contrast-enhanced CT is sensitive to detect tumors smaller than 1 cm. The typical characteristic of TJP on MRI is “salt and pepper” appearance which is most apparent in tumors greater than 1 cm [35](Figs. 2 and 3). The “salt” is blood products from hemorrhage and the “pepper” is flow voids due to high vascularity. The sensitivities and specificities of contrast-enhanced MRI for detecting HNPGLs are 90–95% and 92–99%, respectively [36]. Angiography is valuable to demonstrate multiple enhancing, feeding peri-tumoral vessels, and both CTA and MRA are useful for revealing multicentric disease [37]. The sensitivity and specificity of contrast-enhanced MRI combined with contrast-enhanced MRA for detecting HNPGLs are 100% and 94%, which are higher than those of contrast-enhanced MRI alone(94% and 41%) [38, 39].Whole-body CT or MRI (between skull base to pelvis) with or without contrast is helpful for screening for additional primary PCC/PGL tumors in patients with elevated plasma free metanephrines. The sensitivity of whole-body MRI is much higher than that of biochemical testing (87.5% vs. 37.5%) for SDH-related tumors [40]. Contralateral transverse sinus and jugular systems must be evaluated radiologically since an absence or hypoplasia of contralateral venous system may be a contraindication for surgery due to a high risk of venous stroke after surgery [41]. Other imaging techniques such as dynamic contrast-enhanced MRI perfusion, MR spectroscopy, and nuclear medicine functional imaging are also useful for diagnosis of TBPs [42••, 43–45].

### Preoperative embolization

**Fig. 4:**
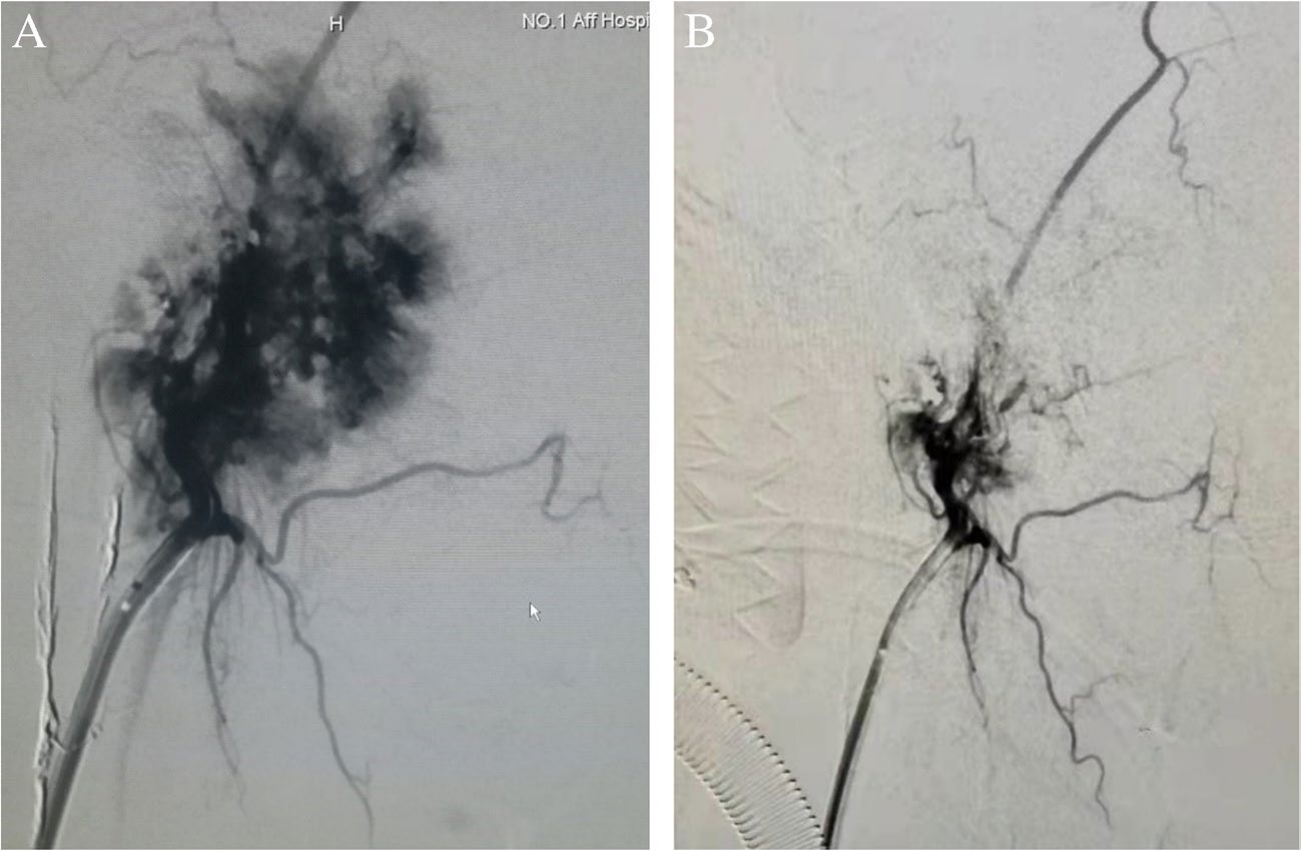
**A** DSA prior to embolization showing a highly vascularized TJP. **B** DSA post embolization showing significant reduction in vascularity. (Same case as in Fig. 3).

Preoperative embolization is usually recommended for Fisch C and D paragangliomas considered for surgery as embolization reduces blood loss and operative time, thereby improves visualization, reduces morbidity, and increases the probability of complete resection [46–48], with low rates of complications such as transient facial pain, blindness, cranial nerve palsies, and stroke [47, 49]. Feeding vessels are usually embolized superselectively 24 to 48 h prior to surgery, following evaluation of tumor blood supply with digital subtraction angiography (Fig. 4), to prevent revascularization and formation of collateral arterial channels. Ascending pharyngeal artery, occipital artery, and posterior auricular artery are the main feeding vessels of TBPs [50, 51]. However, there are controversies on criteria and advantages of embolization yet [52].

### Audiometry and laryngoscopy

The most common symptom of TBPs is hearing loss which presents in 60–80% of cases. Audiometry is necessary in order to objectively document the degree and nature of hearing loss. Hearing loss is usually conductive due to potential mass effect on the ossicular chain and/or tympanic membrane, or from a secondary middle ear effusion. Once the inner ear, internal auditory canal, or cerebellopontine angle are invaded, the patients may have sensorineural or mixed hearing loss.

Laryngoscopy is an essential examination for TBPs since it can document if there is vocal cord paresis indicating lower cranial nerve deficits which usually develop as a consequence of the progressive invasion of the medial wall of the jugular fossa or as a postoperative complication. Sanna et al. have shown that hoarseness is the most common non-otologic symptom which is present in 26.4% of cases, and that the most common CN involved is the IX and X CN each in 37.7% of patients [19]. On the other hand, new postoperative nerve deficits in the 9th and10th CN were reported in 26–42% and 13–28% of cases, respectively [53].

## Treatment

The goal of treatment for TBPs is to achieve tumor control while simultaneously preserve critical neurovascular functions. To date there is no widely accepted algorithm for management of TBPs. Surgery, radiotherapy, watch and wait, or a combination therapy are the usual treatment options. Proposed treatment must be individualized to each patient. During the past decades, surgical management has been the main treatment option for TBPs. However, with recent advances in tumor biology and therapeutic radiology, it seems there is a trend toward a more conservative management such as radiotherapy or active surveillance. Decision-making should take into consideration multiple critical factors such as patient’s age, comorbidities, tumor type and stage, multicentricity, the functionality of cranial nerves, hearing status, venous drainage of the brain, and the degree of carotid involvement [54, 55].

### Surgery

Surgical treatment of TBPs is challenging due to the local anatomical complexity of temporal bone and skull base, and thus the requirement of particular surgical skills and experiences. The indications for resection include young age, catecholamine secreting tumors, significant intracranial pressure symptoms, tumor progression after radiation, facial paralysis, malignant transformation, and lower cranial nerve palsy at presentation [11, 55, 56].

Surgery has been the treatment of choice for most young patients with intact CN function. However, infiltration of the medial wall of the jugular bulb usually indicates a poor prognosis for preservation of LCNs. Another option is to allow the tumor to gradually paralyze the LCNs and then treat surgically after compensation occurs, especially in cases with little probability of neural preservation. In case of elderly patients with normal LCN, surgery is relatively contraindicated since compensation following acute nerve palsies is particularly difficult. Radiological follow-up and RT are usually used for patients older than 60 years and those with poor general condition. Cervical-to-petrous ICA saphenous vein bypass grafting, permanent balloon occlusion, and intra-arterial reinforcement with stenting are options to manage the involved ICA [12]. A large intradural extension (class Di2 TJPs) requires a two-staged procedure to avoid the risk of postoperative cerebrospinal fluid leak resulting from the wide neck exposure [12]. Neck dissection is indicated in metastatic diseases with evidence of lymph nodes involvement at levels I–III[55].

The Fisch A and B paragangliomas are traditionally removed via transmastoid approach or transcanal approach microscopically. Nevertheless, with the advancement of endoscopic otology, transcanal endoscopic approach has been increasingly demonstrated to be a safe and effective technique for the management of middle ear PGs without involvement of mastoid (Fisch A1, A2, and B1) [57–59]. The advantages of endoscopic surgery include low rate of postoperative complications, short hospitalization, and high rate of gross total resection. The Fisch C and D paragangliomas are most commonly treated via infratemporal fossa approach type A and the addition of extensions to it [14, 60]. Class Di3 is preferred to palliative radiotherapy other than surgical management [61, 62].

Although large TBPs (class C and D) can be surgically treated, total grass resection is usually challenging due to the close proximity of tumor to surrounding critical neurovascular structures and thus the high risk of postoperative complications. Subtotal resection is indicated for massive involvement of ICA in cases in which the artery cannot be sacrifice or be stented, and for the elderly patient with tumor adherent to normal LCNs while a piece of tumor can be left over the nerves to avoid postoperative deficit [55]. In a word, surgical management is indicated for TBPs at early stages (classes A and B) with high control rate and less CN deficits compared to larger tumors, and radiotherapy or combination therapy (subtotal resection+radiotherapy) is suggested for tumors at late stages (classes C and D) because of lower complication rates and similar or better local control rates when compared to the surgery [63•, 64].

Numerous reports have shown that postoperative complications such as CN deficits, injuries of carotid arteries and jugular vein, stroke, and cerebrospinal fluid leak are inevitable. CN VII, IX, and X are the most frequently affected nerves[54, 65]. Therefore, the risk of CN impairments must be taken into consideration preoperatively. The patients with CN deficits present with dysfunctional swallowing, disturbed vocal cord function, paralysis of the tongue, and aspiration. In some cases, nasogastric feeding tube, temporary tracheostomy, and percutaneous gastrostomy may be needed. The decision whether to remove the tumor totally and to preserve the nerve’s function is made based on the factors such as the natural history of the tumor, the age of the patient, and the physical condition of the patient.

### Radiotherapy

In the past decades, radiotherapy including stereotactic radiosurgery (SRS), intensity-modulated radiation therapy (IMRT), and proton therapy has been becoming increasingly accepted as a primary or combined treatment of choice for TBPs. The indications for radiotherapy include refusal of surgery, significant comorbidities, preoperative intact LCN function in elderly patients, following planned subtotal removal in C4 tumors, and carotid artery involvement with insufficient collateral vessels in which stenting is impossible and a planned subtotal resection has been performed [55].

In young patients, however, radiosurgery is not usually advocated. Radiotherapy does not cure tumors but only achieves tumor control or volume reduction by approximately 10 to 25%. There are controversies on the long-term effect of radiotherapy to date. It is uncertain whether there is a regrowth of tumor many years after radiosurgery. Prasad et al. have shown that the efficacy of radiotherapy is comparable to that of wait-and-scan [66]. Radiotherapy therefore, is not recommended as curative treatment for large, functional, and/or symptomatic tumors that may be potentially treated by surgery. In addition, radiotherapy is not indicated for patients with significant intracranial extension since it may cause cerebral edema and raise intracranial tension. Staged resection is the treatment of choice for such cases.

SRS includes Gamma Knife, CyberKnife, and linear accelerator. Although there is no statistically significant difference among the SRS techniques, Gamma Knife is the most commonly used radiosurgery modality with high tumor control rate [67–69]. Shapiro et al. showed that the tumor control rate, symptom control, and complications rate following primary radiosurgery (PRS) were 92%, 93%, and 8%, respectively, which indicates that PRS is safe and effective at controlling growth and clinical symptoms of glomus jugulare tumors [70]. Studies have shown similar control rates and potentially lower complication rates of SRS for TBPs as compared with surgery, and comparable tumor control between SRS and EBRT [53, 71, 72]. Ivan et al. [69] showed that patients undergoing SRS had the lowest rates of recurrence compared to subtotal resection (STR), gross-total resection, and STR+SRS and that patients who underwent gross total resection sustained worse rates of cranial nerve deficits with regard to CNs IX–XI than those who underwent SRS alone. Patel et al. have shown excellent 5-year (98%) and 10-year (94%) tumor control in glomus jugulare tumors treated by SRS, and the tumor control rate drops to 74% at 15-year follow-up [73].

Rougier et al. [74] have shown that IMRT at a dose of 45 Gy in 25 fractions achieves 100% of local control rate, which indicates that IMRT is an efficient and safe treatment for HNPGLs with a low toxicity profile and excellent local control. Proton beam therapy is another effective and well-tolerated treatment modality for skull base paragangliomas [75].

Common complications of radiotherapy include skin erythema, xerostomia, mucositis, and nausea. A few patients have bone or brain necrosis, dysphagia, or cranial nerve deficits. In addition, a risk of inducing malignancy by irradiation over a long period of time has been reported [72, 76].

### Chemotherapy

Conventional chemotherapy with cyclophosphamide, vincristine, and dacarbazine has been reported for metastatic diseases with complete or partial tumor response rate of between 4 and 37% [77]. The response to chemotherapy agents in paragangliomas with germline SDHB mutations is better than that in sporadic diseases [78]. In addition, recent studies have shown promising outcomes of peptide therapy and immunotherapy for treatment of metastatic disease [79, 80].

### Watch and wait

HNPGLs are indolent benign tumors and grow slowly with a rate of 0.8–2mm/year, and grow more slowly in patients over age 50 [81, 82]. Tamaki et al. reported that tumor doubling time was between 6 months and 21.5 years and that over half of the observed paragangliomas had no growth during the time of observation [83•]. Prasad et al. [66] demonstrated that 65% classes C and D remained stable or even regressed in size over a median follow-up of 61 months. In addition, a wait-and-scan approach allows for determination of tumor stability before an intervention is determined. Therefore, observation and active surveillance are recommended increasingly for selected cases such as older patients, patients with serious medical comorbidities, multiple tumors, or those with a high risk of postoperative cranial neuropathy due to the possible short- and long-term morbidities following surgery and/or radiotherapy [66, 84].

### Follow-up

Although the risk of recurrence following treatment in HNPGLs is less than 10%, the recurrence rate is higher in those with familial disease, and the median time to recurrence in HNPGLs is 5.1 years [85••]. Therefore, no matter what treatment regimen is selected, patients should be followed up closely with clinical examination, laboratory test, and repeat imaging. Clinical examinations include cranial nerve examination and audiometry. Biochemical testing of plasma or urinary metanephrines should be done every year in patients with elevated preoperative metanephrines or with high risk of recurrent or metastatic disease. MRI is the usual imaging modality for post treatment follow-up. The British Skull Base Society recommends yearly imaging for the first 3 years with reduced follow-up intervals thereafter [86]. The European Society of Endocrinology recommends postoperative follow-up for at least 10 years in all patients to monitor local or metastatic recurrence or new tumors [33].

## Summary

TBPs are rare and typically benign tumors of temporal bone. Management of TBPs requires a thorough understanding of pathophysiology of the tumor including the biochemistry, genetics, and metastasis. Surgery, radiotherapy, and active surveillance are treatment options, and should be individualized to patients based on multiple factors. Multidisciplinary team consisting of neurotologist, interventional neuroradiologist, neurosurgeon, endocrinologist, radiation oncologist, geneticist, and radiologist can help to maximize curative effect and minimize occurrence of complications.

## Data Availability

Not applicable

## Abbreviations

CN: Cranial nerve
DSA: Digital subtraction angiography
HNPGLs: Head and neck paragangliomas
ICA: Internal carotid artery
LCN: Lower cranial nerve
PGLs: Paragangliomas
PCCs: Pheochromocytomas
PPGLs: Pheochromocytomas and extra-adrenal paragangliomas
SRS: Stereotactic radiosurgery
SDH: Succinate dehydrogenase
TBPs: Temporal bone paragangliomas
TJPs: Tympanojugular paragangliomas
TMPs: Tympanomastoid paragangliomas
